# Nuclear blindness: An overview of the biological weapons programs of the former Soviet Union and Iraq.

**DOI:** 10.3201/eid0504.990408

**Published:** 1999

**Authors:** C J Davis

**Affiliations:** Johns Hopkins University Center for Civilian Biodefense Studies, USA. christopher.davis1@virgin.net


### Nuclear Blindness: An Overview of the Biological Weapons Programs of the Former Soviet Union and Iraq

**Christopher J. Davis**
Johns Hopkins University Center for Civilian Biodefense Studies

The demise of the biological weapons capability of the United States in 1969 and the advent of the Biological and Toxin Weapons Convention in 1972 caused governments in the West to go to sleep to the possibility of biological weapons development throughout the rest of the world, as technically knowledgeable workers were transferred and retired, intelligence desks were closed down, and budgets were cut. By 1979, despite the Sverdlovsk anthrax release, a senior British government policy official described any biological weapons threat as nebulous. President Nixon's biological weapons disarmament declaration in 1969 had conveyed the impression that biological weapons were uncontrollable and that the U.S. program had not been successful in producing usable weapons (when in fact the opposite was true). Add to this the rise of truly intercontinental ballistic missile delivery of nuclear weapons, and the stage was set for what I have termed "nuclear blindness" and defined as "the tunnel vision suffered by successive governments, brought on by the mistaken belief that it is only the size of the bang that matters." Throughout this period, both the former Soviet Union and Iraq conceived, albeit in different ways, their new biological weapons programs. It took until 1989-1991 for government technical experts in the West to persuade the world and their own governments that these programs were real and of enormous potential importance to the security of the West, if not the whole world.

Too many times in the past we have failed to anticipate future developments; refused to think the unthinkable and expect the unexpected. Too many times we have been out maneuvered by those who take the time to think and plan and do not simply rely on reacting to events. We must learn to think like our potential adversaries if we are to avoid conflict or blunt an attack, because only superior thinking and planning (not just better technology) will enable us to survive biological warfare.

Address for correspondence: Christopher J. Davis, Og House, Ogbourne St. George, Marlborough, Wiltshire, SN8 1TF, United Kingdom; fax: 44-(0)-1672-841418; e-mail: christopher.davis1@virgin.net.

### The Former Soviet Union

The origins of the biological weapons program of the former Soviet Union stretch back to statements by Lenin, and experimental work was under way by the late 1920s. The modern era was ushered in, however, only with the postwar military building program, which established infrastructure for research, development, testing, production, and delivery of a variety of agents and weapons.

On the other side of the globe, the allied biological weapons program had grown from the fledgling efforts of British research into anthrax and the development of the World War II–anthrax cattlecake retaliation weapon into a large U.S.-based research and development (R&D) and production capability. By 1969, the U.S. military had accepted seven type-classified agents, and, at plants such as the one at Pine Bluff in Arkansas, they could produce 650 tons of agent per month for filling into weapons. This thriving offensive program was unilaterally abandoned in 1969 as a result of a complicated mixture of politics, secret intelligence information, new technological developments, and the Vietnam War. These developments gave impetus to the creation of the Biological and Toxin Weapons Convention, originally drafted by the British but finalized by the Soviet Union. Although the Soviet Union signed the Convention at its inception in 1972, it did not believe that the United States would be so foolish as to abandon its biological weapons capability, regarding the disarmament agreement as a 'worthless piece of paper.'

In 1973 and 1974, the Soviet Politburo formed and funded the organization known most recently as Biopreparat (Chief Directorate for Biological Preparations), designed to carry out offensive biological weapons R&D and production concealed behind legal and civil biotechnology research. At no time did civilian biotechnology work ever comprise much more than 15% of the activity at any of the 52 sites under the aegis of Biopreparat. Ultimately it was controlled by the Ministry of Defense, the Military Industrial Commission, and other state organs, all the way up to the Central Committee and what became eventually the Office of the President. Its head, a general, retained special access to the Central Committee from its inception, and through its links with the Academies of Science and Medical Science, Ministry of Health, and the Anti-Plague Institutes, recruited a generation of scientists who elsewhere in the world underpinned the expanding pharmaceutical and biotechnology industries and academic life-sciences research. The whole system probably employed at its height at least 50,000 people, many of whom were scientists and technicians with very high security clearance that identified them as part of a biological weapons program more closely held and more secret than its nuclear weapons counterpart. The system was always able to draw on the best from any source but was, to a certain extent, self-sufficient. Not all of the 52 establishments were occupied with microbiology or weapons—some were workshops, garages, and cover operations; others supported the program directly with fermenter design and construction or building of weapons test chambers; while yet others carried out advanced research, which would then be given to other institutes for development. Often there was internal competition, with one project being given to a number of facilities to see who would come up with the best idea. In its first 15 years alone, Biopreparat probably cost at least 1.5 billion rubles to create and run—a large sum for life-sciences R&D but relatively modest compared with the cost of nuclear weapons R&D and, therefore, in terms of strategic weapons, extremely cost-effective.

The main purpose of the enormous Biopreparat capability was to hide biological weapons research, development, and production formerly carried out solely in Ministry of Defense establishments behind a facade of nominally civilian biotechnology and pharmaceutical enterprises. The two systems, the former Ministry of Defense complex of biological weapons facilities and the new Biopreparat facilities, continued to operate side-by-side. The Ministry of Defense facilities themselves probably employed another 15,000 workers and had a separate budget, so that the potential within the system as a whole, which is how it should be considered, was large and dwarfed the by-then long-abandoned U.S. offensive program. Its capacity for production of agent was measured not in tons but in hundreds of tons for each of at least nine separate sites, primarily plague, tularemia, glanders, anthrax, smallpox, and Venezuelan equine encephalomyelitis.

Another mission of Biopreparat was to apply advances in biotechnology (genetic engineering, in particular) to improving the biological weapons capability of the former Soviet Union. This mission took several forms, supported primarily by the then vice-president of the Academy of Sciences, Yuri Ovchinnikov, the most influential Soviet biomedical scientist of the 1970s. He saw a way around arms control treaties and weapons conventions by using microbes to produce biologically active substances that would replace classic chemical weapons; their production could then be concealed in the biotechnology or pharmaceutical industry. He also envisaged that the government would use genetic engineering to produce a new generation of biological weapons agents with enhanced capability for expressing toxins and other biologically active substances and to improve overall weapons effectiveness. The outcome of the first of these two programs is not known, but the latter was very successful. Moreover, the new Biopreparat-based program was able to address all aspects of agent production and delivery, not just the most advanced microbiological ones. It built strength in depth, having as its main aims to improve industrial production scale-up techniques, microbial production rates, yields of viable microorganisms, virulence, and resistance of microorganisms to antibiotics; to maximize viability of agent during dissemination and increased survivability of biological aerosols; and to enhance the ability of microorganisms to degrade the target's natural defenses. The leaders of the program foresaw increasing encroachment of international arms control processes into the territory of sovereign states. Thus, they perceived the need for its weapons to become invulnerable to first strike or counterattack. Key technical targets associated with such an approach were the development of dry solid particulate agent formulations, miniaturized production facilities, mobile production and filling facilities, strains resistant to multiple antibiotics, cruise missile dissemination system, and combination organisms.

By addressing every aspect of weapon production, from selection of new strains of organisms to the behavior of biological aerosols under every possible condition of climate and topography, through the genetic engineering of antibiotic resistance and the design of optimum dissemination and delivery systems, the former Soviet Union was able to envisage the achievement of a miniaturized mobile production and weapon-making capability invulnerable to clandestine monitoring, invasive arms inspection, or attack in the event of war (because it was beyond identification); agents precisely matched to particular scenarios and human targets and incapable of being treated; a variety of dissemination systems, including cruise missiles; agents resistant to degradation by heat, light, cold, UV radiation, ionizing radiation, and various antibiotics; and dry formulations of agents capable of remaining viable in long-term storage.

By the time of the breakup of the former Soviet Union, from which the Russian Confederation emerged in 1992, much had been achieved and war mobilization plans were in place for the surge production of huge quantities of the agents mentioned earlier, as well as a number of others, such as Marburg virus. Of overwhelming importance has been the capability to undertake a strategic attack using plague or smallpox. Intercontinental ballistic missiles with MIRVed warheads containing plague were available for launch even before 1985, and SS-11 and SS-18 missiles have been mentioned in this connection. Concepts of use had been developed for each of the biological agents formally accepted into use by the army. For instance, the principal agents designated as tactical or operational for use on the battlefield were tularemia and Venezuelan equine encephalomyelitis, whereas anthrax and Marburg virus were nominated for attacking rear areas. The third category of agents comprised the highly transmissible agents smallpox and plague, which were categorized as strategic weapons and destined for use against enemy population centers.

What happened after Vladimir Pasechnik (the former general director of Science Production Organisation Farmpribor and director of The All Union Scientific Research Institute of Ultra Pure Biopreparations in Leningrad [St. Petersburg]) defected in 1989 constitutes a long and complex story, but in January 1991 the first-ever visit to Biopreparat facilities was undertaken, by a joint U.K./U.S. technical team, under a cloak of secrecy. After the subsequent defection of Kanadjan Alibekov (a former senior deputy director of Biopreparat) in 1992, the United States and the United Kingdom were certain enough that the offensive biological weapons program was continuing that they challenged the new Russian regime openly about it as late as 1993. By then substantial changes had taken place within Biopreparat, and today a concerted effort is under way to help the Russians civilianize these former biological weapons R&D establishments. However, questions remain about the Russian program: What happened to the part of the program in Ministry of Defence facilities that western experts have been unable to visit? What happened to plans detailing every aspect of production and deployment? What happened to the Ovchinnikov bioregulator program? What happened to the thousands of personnel involved in the Biopreparat program? What happened to the R&D centered on anticrop, antiplant, and antilivestock biological weapons? What happened to the stocks of seed cultures of biological weapons agents designed to be used to fuel the mobilized production of weapons? Was there space-based biological weapons capability? Was there any human genetics-related biological weapons research?

Despite the passage of nearly 10 years, the fundamental change in political structure of Russia, the extreme economic upheaval and budget restrictions, the reorientation of Biopreparat's work, and the help and support given by the West to civilianize programs and stop the transfer of technology and scientists into illegal biological weapons programs, the capability of the old Russian Ministry of Defence sites remains largely unknown.

### Iraq

Iraq has stated that its biological weapons program dates to at least 1974. It was carried out in great secrecy, after the Biological and Toxin Weapons Convention had been signed. The program was first conducted in an ostensibly civilian organization called the State Organization for Trade and Industry until this was superseded by the Military Industrial Commission. As with all other major military programs, biological weapons R&D was able to call upon many of its leading scientists who undertook undergraduate or postgraduate training in the west. Much of what happened between the supposed inception of the program in 1974 and the establishment of a group of biologists within the Al-Muthanna chemical weapons complex in 1984 is unknown.

In 1987, the Al-Muthanna research group was transferred to the Al-Salman facility, and work was expanded to include the investigation of fungal and antiplant agents; 1988 saw the establishment of the Al-Hakam Factory, an industrial-scale production facility designed to produce anthrax and botulinum toxin for filling into weapons. This project was completed quickly by using equipment from nominally civilian facilities, such as those used to produce vaccines; the factory itself produced biological agent, which was filled into weapons and deployed in late 1990. The program was further expanded in 1990 when viruses were added to the range of agents under development and production capacity was enhanced by the acquisition and integration of civilian biotechnology facilities by the Military Industrial Commission.

According to the Iraqis, the program was terminated in 1991, after the adoption of UN SCR687, and agents, weapons, munitions, and documents were destroyed. However, the United Nations Special Commission (UNSCOM) believes that from 1991 to 1995 Iraq actively preserved biological weapons capability.

### The Agents, Weapons, and Means of Delivery

The UNSCOM belief that three biological agents were filled into weapons is supported by Iraqi statements concerning the filling of munitions and their deployment ready for delivery. For one of these agents, *Botulinum* toxin, UNSCOM also possesses objective evidence; the other two were probably anthrax and *Clostridium perfringens* spores. Approximately 380,000 liters of *Botulinum* toxin were manufactured, along with 84,250 liters of anthrax spores and 3,400 liters of *C. perfringens* spores. In addition, 2,200 liters of aflatoxin were produced. All these figures represent preconcentration totals and may be underestimates. Ricin toxin and the antiplant agents wheat bunt and corn smut were also produced. Camel pox is known to have been under development as well. This disparate list of biological agents, which at first seems to contain substances not previously conceived as potential offensive biological weapons agents, on closer inspection reveals a rationale based on the possession of a multipotent arsenal having lethal, incapacitating, oncogenic, ethnic, economic, terror, and variable time-onset capabilities. In addition, these agents are capable of being used to attack people through the lungs and the skin, as well as with carriers such as triethylamine, CN or CS, or as a toxic coating in fragmentation weapons.

Agents were filled into various weapons for dissemination. By the end of 1990, according to Iraqi statements, 25 SCUD/Al-Hussein missiles were readied for use with biological weapons warheads (each carrying 145 liters of agent) and deployed for action. At least 160 R400 retarded aerial bombs, carrying the distinctive black-stripe identification around them, may also have been filled with 90-liter charges of *Botulinum* toxin and ready for use. UNSCOM has evidence to corroborate the Iraqi claim. The Iraqis also intended to fill R400 bombs with anthrax and aflatoxin. Originally designed and filled with chemical agents, 155-mm shells were also tested with a ricin toxin fill. At least three fuel drop tanks were completely modified and fitted with Venturi mechanisms to facilitate aerosol release, for dispersal of 2,200-liter loads of anthrax and possibly *Botulinum* toxin, using F1 aircraft as the delivery means.

### Postscript

UNSCOM has no confidence that Iraq has abandoned its biological weapons program. The true scale and scope of the Iraqi biological weapons program are, despite all UNSCOM's efforts, still not known.

Dr. Davis is consultant in Pharmaceutical Medicine and Applied Physiology and director of the ORAQ Consultancy Ltd., United Kingdom.

